# Heat tolerance of early developmental stages of glacier foreland species in the growth chamber and in the field

**DOI:** 10.1007/s11258-014-0361-8

**Published:** 2014-07-02

**Authors:** Silvia Marcante, Brigitta Erschbamer, Othmar Buchner, Gilbert Neuner

**Affiliations:** Institute of Botany, University of Innsbruck, Sternwartestrasse 15, 6020 Innsbruck, Austria

**Keywords:** Alpine plants, Germination, Heat tolerance, Risk assessment, Seeds, Temperature stress

## Abstract

In glacier forelands, seeds readily germinate, however, a high proportion of seedlings die shortly after their appearance. We hypothesized that besides drought, frost and missing safe sites, heat on the ground surface could be one of the major threats for seedlings. The heat strain in different ground strata was assessed from 2007 to 2010. The heat tolerance (LT_50_) of eleven alpine species from different successional stages was tested considering imbibed (G1) and germinated seeds (G2) as well as seedlings (G3). Additionally, the heat hardening capacity of seedlings was determined in the field. Across all species, LT_50_ decreased significantly by 9 K from G1 (55 °C) to G3 (46 °C), similarly in all species of the successional stages. Field-grown seedlings had mostly an increased LT_50_ (2K). Intraspecifically, LT_50_ of seedlings varied between 40.6 and 52.5 °C. Along the chronosequence, LT_50_ in G1 was similar, but was higher in G2 and G3 of early successional species. The highest temperatures occurred at 0–0.5 cm in air (mean/absolute maximum: 42.6/54.1 °C) posing a significant heat injury risk for seedlings when under water shortage transpirational cooling is prevented. Below small stones (0–0.5 cm), maxima were 4 K lower, indicating heat safer microsites. Maxima >30 °C occurred at 32.3, >40 °C at 6.2 %. Interannually, 2010 was the hottest year with heat exceeding LT_50_ at all microsites (0–0.5 cm). Temperature maxima on sandy surfaces were lower than on microsites with gravel (diameter <5–10 mm). The hot summer of 2010 may be a small foretaste of in future more severe and frequent heat waves. Ground surface temperature maxima at the pioneer stage are already now critical for heat survival and may partly explain the high seedling mortality recognized on recently deglaciated terrain.

**Electronic supplementary material:**

The online version of this article (doi:10.1007/s11258-014-0361-8) contains supplementary material, which is available to authorized users.

## Introduction

Maximum free air temperatures are too low to cause heat damage to plants above the treeline in temperate-zone mountains. However, alpine plants often show a prostrate growth that causes a decoupling from ambient air temperature. Depending on plant stature, plant body temperatures can exceed air temperature by 20–30 K (Salisbury and Spomer 1964; Körner and Cochrane 1983; Gauslaa 1984; Körner 2003; Larcher and Wagner 2009, 2010; Larcher 2012; Neuner and Buchner 2012). The maximum plant body temperatures found during summer in the field actually come close to the observed heat tolerance of alpine plant species (Körner 2003). Vegetative above-ground plant parts of alpine species get damaged by heat between 42 and 64 °C (Neuner and Buchner 2012) which indeed has occasionally been observed to occur naturally at alpine field sites (*Loiseleuria procumbens* and *Carex firma,* Larcher and Wagner 1976; *Saxifraga paniculata,* Neuner et al. 1999; *Minuartia recurva,* Buchner and Neuner 2003; *Silene acaulis,* Gauslaa 1984; Körner 2003). Particularly, in prostrate plants growing in isolation or with large spacing in shallow soil during dry periods with full sunshine, the risk of damage by heat is increased. On bare spots without evaporative cooling, dry soil surfaces can heat up in extreme even to 80 °C near the alpine timberline (Turner 1958; Körner and Cochrane 1983) and patches of bare humic soil within alpine vegetation appeared likely to be related to heat stress (Körner 2003).

While heat tolerance of adult alpine plants of vegetative above-ground organs (reviewed by Neuner and Buchner 2012) and reproductive structures (Ladinig, Neuner and Wagner unpubl.) in adult alpine plants is well understood, hardly any information exists about the heat susceptibility of germinating seeds and early developmental stages (seedlings and juvenile plants) and of vegetative dispersal units such as plantlets or bulbils. To our knowledge, only a preliminary study has addressed this question to some extent. This study, investigating four alpine plants (Wildner-Eccher and Larcher, unpublished; cited in Neuner and Buchner 2012), suggests significant differences to the heat tolerance of vegetative parts of adult plants: (1) early developmental stages having a tentatively lower heat tolerance than adults; (2) dry seeds showing a significantly increased heat tolerance compared to vegetative shoots of adults but being unable to survive peak soil surface temperatures (cf. 80 °C) reported for the respective altitudes of the Central European Alps.

In the glacier foreland on recently deglaciated terrain, germination and establishment of young plants take place on bare ground. Although a considerable number of seeds readily germinates (Niederfriniger Schlag and Erschbamer 2000; Marcante et al. 2009a), establishment occurs only at very low rates as seedling mortality is high (Niederfriniger Schlag and Erschbamer 2000; Welling et al. 2005; Erschbamer et al. 2008; Marcante et al. 2009a). While the low frost resistance during and after germination may at least in certain years explain the high seedling mortality rates (Marcante et al. 2012), also other abiotic and/or biotic factors must play an important role in this respect (Matthews 1992; Erschbamer et al. 2008; Marcante et al. 2009a). One possible explanation for the high seedling mortality rates could be heat stress perceptible on the surface of bare ground. During short dry periods within the growing season, these heat effects will additionally get aggravated when cooling by transpiration is reduced. Heat waves such as that in 2003 (García-Herrera et al. 2010) seriously affect plants growing at high altitudes (Abeli et al. 2012) influencing seed persistence and germination (Walck et al. 2011). On bare ground surfaces of a glacier foreland, we expect that critically high heat events may occur also in ‘common’ summers, but will be even pronounced in heat wave years (Barriopedro et al. 2011).

Survival of abiotic stress may highly depend on the availability of specific microsites and the presence of safe sites (Choler et al. 2001; Callaway et al. 2002; Kikvidze et al. 2005; Callaway 2007). Soil particle sizes (Chambers et al. 1991) and microrelief govern seedling recruitment in the glacier foreland (Niederfriniger Schlag and Erschbamer 2000; Erschbamer et al. 2008). We hypothesize that these parameters control also the temperature pattern at the ground surface and thus enhance or lower the heat stress for the seedlings.

In the present study, we investigate heat tolerance of imbibed seeds, germinated seeds and seedlings of glacier foreland plants, comparing pioneers, early and late successional species. By linking heat tolerance with temperature maxima in their habitat for the years 2007–2010, the potential risk of suffering heat damage during different developmental stages should be assessed. We addressed the following questions: (1) are there differences in the heat tolerance of glacier foreland species and which organs are the most susceptible ones? (2) What is the maximum air temperature a seedling experiences in the pioneer stage of the glacier foreland and do seedlings have a heat hardening capacity in the field? (3) How does the microsite affect maximum air temperature at the ground surface? (4) At what frequency may early developmental stages get exposed to high air temperatures in the glacier foreland and at what probability will they suffer from heat damage under water shortage?

## Materials and methods

### Plant material

Eleven alpine plant species (Table 1), all of which occur along the primary succession of the Rotmoos glacier foreland (Obergurgl, Ötztal, Tyrol, Austria, 46°49′N 11°02′E), were selected. In 2008, per species, almost 500 seeds were collected at the distribution centre of the species (according to the species scores in the CCA plot, Schwienbacher et al. 2012) along the chronosequence (Table 1), i.e. on the pioneer stage (moraine 1971), early successional stage (moraine 1923) and on the late successional stage (moraine 1858). For detailed site descriptions, see Raffl and Erschbamer (2004) and Raffl et al. (2006). Prior to the heat tolerance test, seeds of all species were sown into Petri dishes and cultivated in a growth chamber (Sanyo, E&E Europe BV, Leicestershire, UK; MLR-350H; 25/10 °C, 16/8 h light/dark, 400 µmol photons m^−2^ s^−1^) to the respective development stages.

**Table 1:** Occurrence of the eleven investigated alpine plant species on three successional stages (pioneer, early and late) of the Rotmoos glacier foreland deglaciated since 1971, 1923, 1858, respectively, and abbreviations used in the text

Successional stage	Plant species	Abbreviation
Pioneer	*Saxifraga aizoides*	SAXAIZ
	*Artemisia genipi*	ARTGEN
	*Oxyria digyna*	OXYDIG
	*Arabis coerulea*	ARACOE
Early	*Trifolium pallescens*	TRIPAL
	*Persicaria vivipara*	PERVIV
	*Geum reptans*	GEUREP
	*Poa alpina*	POAALP
Late	*Leontodon hispidus*	LEOHIS
	*Achillea moschata*	ACHMOS
	*Silene acaulis* agg.	SILACA

The nomenclature follows Fischer et al. (2008)

To test the natural heat tolerance, seedlings were additionally germinated at the research site on the 1971 moraine (i.e. pioneer stage, 2,400 m asl), being ideal due to the presence of large bare ground areas and due to the possibility of artificial irrigation (Schwienbacher et al., unpubl.). In autumn 2008, two bare ground plots were prepared with the natural substrate characteristic for this successional stage. A total of 100 seeds per species per plot were sown in rows of 5 cm width (one row per species), the row being separated by 10 cm. To prevent drought stress to seedlings and to ensure low mortality rates, the plots were irrigated daily twice by a self-constructed automatic irrigation system using spring water. This should ensure availability of sufficient field-grown seedlings for the experiments in 2009. Bulbils of *Persicaria* *vivipara* were regarded as seeds, as they occur in the seed bank of the Rotmoos glacier foreland (Marcante et al. 2009b). To prevent drought stress to seedlings and to ensure low mortality rates, the experimental field was regularly watered during the 2009 growing season.

### Developmental stages

The following development stages were investigated: seeds (G1, imbibed seeds), germinated seeds (G2, emerged radicle at least 2 mm long) and seedlings (G3, cotyledons completely expanded). To test heat tolerance of germinated seeds and seedlings, seeds were germinated in the growth chamber under standard conditions (25/10 °C, 16/8 h light/darkness, 400 µmol photons m^−2^ s^−1^) and then cultivated in the laboratory.

### Microclimate

Throughout four successive summer periods (2007–2010), a microclimate station was operated at the pioneer stage of the Rotmoos glacier foreland (moraine 1971, 2,400 m asl). At the microsites where seedlings establish, temperatures were recorded in order to assess the frequency, duration and severity of heat events. Temperature sensors were positioned at random within an area of 60 m^2^ at the pioneer stage of the Rotmoos glacier foreland on a plain, bare ground site. Micropositioning of the temperature sensors were chosen such as the sensors were placed on bare ground surfaces close to the artificial irrigation experiment carried out from 2007 till 2010 on this site (Schwienbacher et al. unpubl., FWF-project P19090-B16). Air temperatures (1 cm above the ground surface; five sensors), ground surface temperatures (0–0.5 cm; sensor tip in air: 14 sensors; sensor tip below small stones: six sensors) and soil temperatures (−0.5 cm; two sensors) were measured with thermocouple sensors (Type T, solder junction diameter: 0.2 mm, TT-Ti 36, Omega, Stamford, USA). For ground surface temperatures (0–0.5 cm), the grain size at the microsite was measured and allocated to three grain size classes: <2 mm (sand), <0.5 and <1 cm. Soil temperatures at lower depths than −0.5 cm (−2, −4 and −10 cm) were additionally recorded with thermistors (107, Campbell Scientific, Logan, UT, USA). All sensors types were connected to a data logger (CR10X, Campbell Scientific) that was programmed to collect data every 30 s and to record mean values at 10 and 30 min intervals.

### Heat tolerance

Heat treatment to test heat tolerance was conducted in hot water baths (Thermomix Braun, Melsungen, Germany). All developmental stages were exposed for a duration of 30 min, to a set of heat temperatures differing by 2 K ranging between the temperature at 0 and 100 % heat damage (36–60 °C). At each exposure temperature, ten replicates of each developmental stage were tested for heat tolerance. The heat tolerance of the seedling organs (roots, hypocotyl and cotyledons) was tested separately at each exposure temperature on three to five seedlings. The samples were placed on wet filter paper and plunged inside heat-durable and watertight plastic bags into the preheated water baths to bring them immediately to the exposure temperature as is standard in heat tolerance tests (Kreeb 1990). After a period of 5 days (exposure in the growth chamber), when heat damage symptoms had developed, viability of the samples was assessed. Viability was checked for the developmental stage samples as a whole and for single organs by a tetrazolium test (TTC). By this test (Ruf and Brunner 2003; Larcher et al. 2010; Marcante et al. 2012), dehydrogenase activity reduces the colourless tetrazolium salt to red-coloured triphenyl formazan, so that red-coloured cells and organs can be rated as viable (Larcher 1969).

The percentage of heat damage was calculated with image analysis software (OPTIMAS, 4.5, Media Cybernetics, Rockville, MD, USA) and then plotted against the treatment temperature. A classic logistic function was fitted to the data with P-Fit software (Biosoft, Durham, NC, USA):

$$ y = {\text{Min}} + \frac{{{\text{Max}} - {\text{Min}}}}{{1 + e^{ - k(X - X50)} }} $$

where *X* is the input variable (target temperature), *Y* is the output variable (injury as a percentage), Min and Max are asymptotic upper and lower limits of the curve (0, 100 %), *X*50 is the input variable at the inflection point (Min − Max)/2, and *k* is a slope factor. Values of *X*50 were read directly from the fitted curve and used as a measure of heat tolerance (i.e. taken as LT_50_, the lethal temperature (°C) for half of the samples).

### Statistical analysis

Statistics were performed using STATISTICA 6.1 (Stat. Soft Inc., Tulsa, OK, USA) and IBM SPSS STATISTICS (version 21, IBM, USA). Differences of heat tolerance (LT_50_) between developmental stages (G1, G2 and G3), between species and between successional stages were assessed by the multivariate nonparametric Kruskal–Wallis ANOVA test. The heat hardening capacity of seedlings, field-grown versus laboratory grown seedlings, was tested with the nonparametric Mann–Whitney *U* test.

Temperature maxima (half an hour means) were tested for normal distribution by the Shapiro–Wilk test. The homogeneity of variances was examined by the Levene test. Then, the effects of year, micropositioning of temperature sensors and grain size at the measurement spot on maximum temperature (half an hour means), were analysed by three-way ANOVA and the Duncan post hoc test.

Box plots show the median, the 25 and 75 % quartiles, respectively, given by the lower and upper edges of boxes. Whiskers show the 95 % confidence interval. Outliers are not shown.

## Results

### Heat tolerance of early developmental stages grown in the growth chamber

**Fig. 1:**
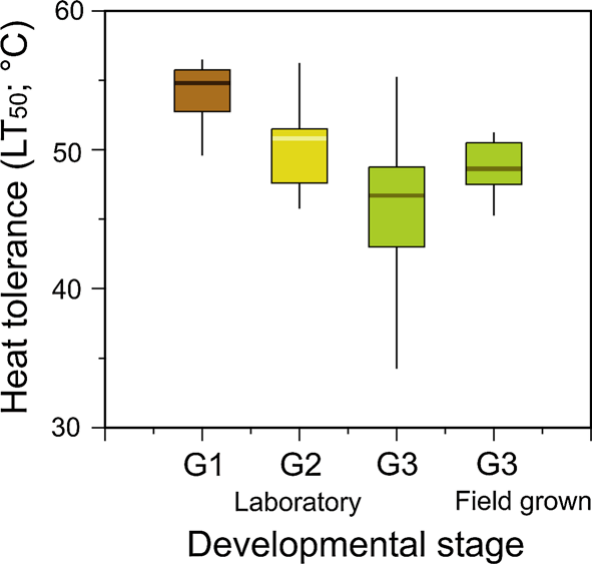
Decrease in heat tolerance (LT_50_ °C) during germination in a growth chamber from imbibed seeds (*G1* *brown*) to germinated seeds (*G2* *yellow*) and seedlings (*G3* *green*) as compared to field-grown seedlings determined for all investigated species from the Rotmoos glacier foreland

Across all species (Fig. 1), seeds (G1) were the most heat tolerant developmental stage followed by germinated seeds (G2) and seedlings (G3; H(2, 96) = 55.2, *p* < 0.001). During germination, the median LT_50_ decreased significantly by 4 K from 55 °C in seeds to 51 °C in germinated seeds and by 9 K to 46 °C in seedlings (see also Supplement 1).

**Fig. 2:**
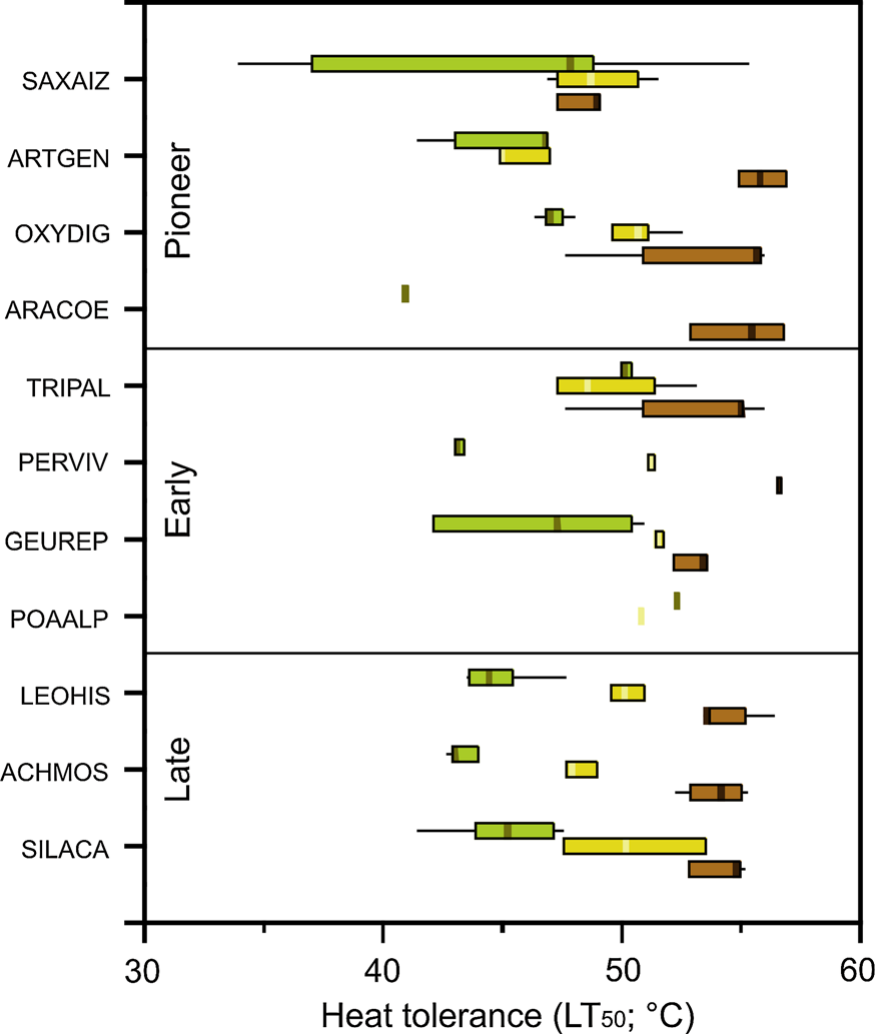
Variability of heat tolerance (LT_50_; °C) in different alpine plant species from the Rotmoos glacier foreland during early development: imbibed seeds (*G1* *brown*), germinated seeds (*G2* *yellow*) and seedlings (*G3* *green*)

The mean heat tolerance of the seeds varied between 49.3 °C for *Saxifraga aizoides* and 56.5 °C for *Persicaria vivipara* bulbils followed by *Arabis coerulea* and *Artemisia genipi* (55.1 and 55.9 °C, respectively) that of germinated seeds between 44.8 °C for *Artemisia genipi* and 51.5 °C for *Geum reptans*. Among the seedlings, those of *Arabis coerulea, Achillea moschata* and *Persicaria vivipara* were the most susceptible to heat (40.6, 43.1 and 43.4 °C, respectively), while those of *Trifolium pallescens and Poa* *alpina* were the most heat tolerant ones (50.2 and 52.5 °C, respectively, Fig. 2).

The heat tolerance of all three developmental stages was relatively uniform only within the late successional species group, showing a general trend. Considerable variations were recognizable within the early successional species as well as within the pioneer group, following no overall trend. Among the pioneer seeds, *Saxifraga aizoides* exhibited the lowest heat tolerance (mean = 49.3 °C) and *Artemisia genipi* the highest (55.9 °C, Fig. 2). The bulbils of *Persicaria vivipara* were the most tolerant ones within the early successional species (56.5 °C), being more tolerant than the seeds of the late successional species (mean = 54.2 °C). Among the pioneer seedlings (G3), *Arabis coerulea* was the most susceptible species (40.6 °C), whereas *Oxyria digyna* was the most tolerant one (46.9 °C). Seedling heat tolerance of the early successional species varied between 43.4 °C (*Persicaria vivipara*) and 52.5 °C (*Poa alpina*), that of the late successional species between 43.1 °C (*Achillea moschata*) and 45.2 °C (*Silene acaulis*).

**Fig. 3:**
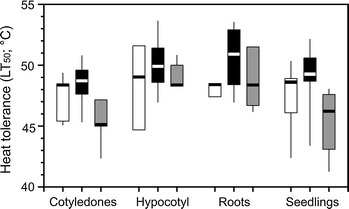
Heat tolerance (LT_50_; °C) of different organs of seedlings of species from the Rotmoos glacier foreland found at the pioneer (*white*), early (*black*) and late (*grey*) successional stages

In the seedling stage, significant differences in heat tolerance between different organs (cotyledons, hypocotyl and roots) were observed (KW-H(4, 259) = 27.1, *p* < 0.001, Fig. 3). Across all species, the cotyledons turned out to be the most heat susceptible organ (47.5 °C), being significantly different (*p* < 0.01) from roots (49.0 °C) and hypocotyl (49.5 °C). Considering the species along the successional sequence, cotyledons of the pioneer and the early successional species had a higher heat tolerance compared to that of the late successional species (KW-H(2, 45) = 12.8, *p* < 0.01). At the species level, the pioneer species *Artemisia genipi*, *Arabis coerulea* and *Saxifraga aizoides* showed the same trend with a higher heat tolerance of roots and hypocotyl compared to cotyledons and seedlings as a whole. Within the early successional species *Trifolium pallescens* and *Poa* *alpina*, the hypocotyl was the most heat tolerant organ (*p* < 0.01), while *Persicaria vivipara* roots were significantly (*p* < 0.01) more heat tolerant than hypocotyl and cotyledons. Within the late successional species, *Silene acaulis* seedlings and cotyledons had a mean heat tolerance of 47.2 °C, while the roots tolerated up to 51.5 °C (*p* < 0.01).

### Heat tolerance of seedlings grown in the field

Across all investigated glacier foreland species, median heat tolerance (LT_50_) of the seedlings grown in the field was 48.8 °C (see Fig. 1), being 2 K higher than that of seedlings cultivated in the growth chamber (46.8 °C; KW-H(1, 113) = 44, *p* < 0.001). Within the pioneer species, the heat tolerance of the seedlings grown in the field was significantly higher (with exception of *Saxifraga aizoides*) than that of seedlings grown in the growth chamber (KW-H (1-55) = 16.2, *p* < 0.01). Within the early successional species, only the seedlings of *Persicaria vivipara* grown in the field turned out to be more tolerant than those grown in the growth chamber (KW-H(1-12) = 6.2, *p* < 0.05). In contrast, seedlings of *Poa alpina* grown in the field showed a lower heat tolerance than those grown in the growth chamber (50.7 and 52.5 °C, respectively, KW-H(1-16) = 11.5, *p* < 0.001). Seedlings of the late successional species *Achillea moschata* and *Silene acaulis* grown in the field had a 3.2–2 °C higher heat tolerance than those grown in the growth chamber.

### Temperature conditions for germination at the field site

**Fig. 4:**
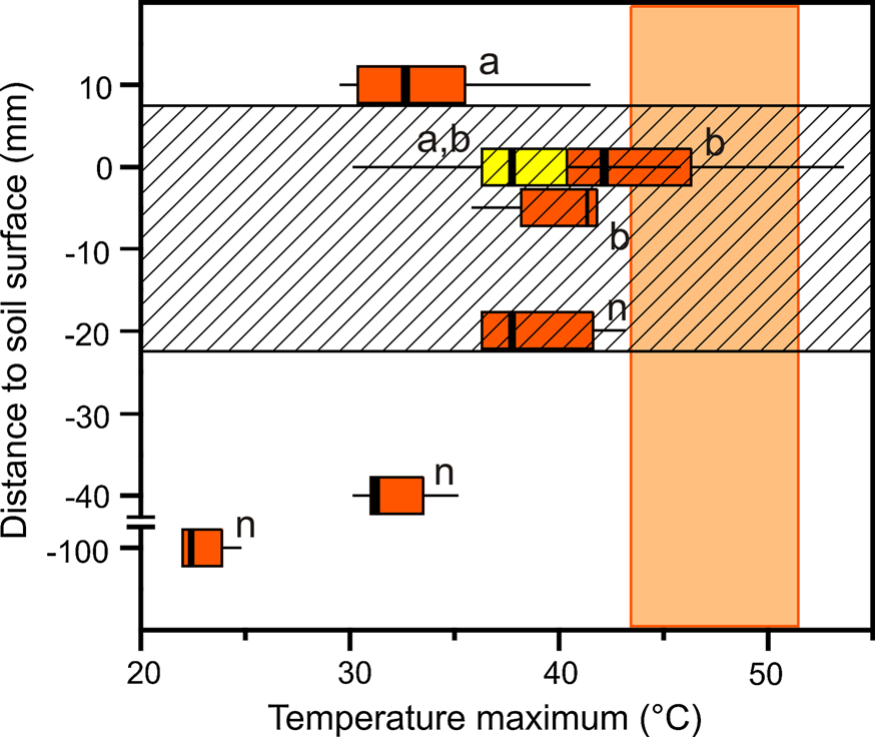
Maximum temperatures (half an hour means) recorded at the ground surface (0–0.5 and 1 cm), on the ground surface, below small stones (0–0.5 cm; *yellow bar*) and in the soil at a depth of −0.5, −2, −4 and −10 cm during summer (June–July–August) of four successive years (2007–2010) at the pioneer stage of the Rotmoos glacier foreland (2,400 m asl). Vertical light orange bar gives the range of heat tolerances (LT_50_) of the investigated field-grown seedlings. The horizontal hatched bar indicates the strata where seedlings establish. *Different letters* indicate significant differences (*n* … not tested)

At the pioneer stage of the Rotmoos glacier foreland (2,400 m asl), the highest heat load was observed in the strata where seedlings germinate and establish (Fig. 4). During the 2007–2010, growing seasons highest maximum temperatures (half an hour means) were recorded in air close to the ground surface (0–0.5 cm). Temperature maxima were at mean 42.6 °C. Maximum temperatures reached and occasionally exceeded heat tolerance determined for field-grown seedlings. In contrast, ground surface temperatures recorded at the ground surface (0–0.5 cm) with the sensor tip covered by a small stone (yellow bar in Fig. 4) were considerably mitigated. Temperature recordings at such microsites reveal approximately 4 K reduced maximum temperatures, i.e. 38.7 °C. This indicates large differences between microsites at the ground surface with respect to the maximum heat load. As like, 1 cm above the ground surface in air, temperature maxima (34.0 °C) were too low to cause heat damage to seedlings. Also at −0.5 and −2 cm temperature maxima were too low for heat damage to the roots of seedling (Fig. 4). With increasing soil depth heat became successively reduced down to a moderate value of 23.1 °C at −10 cm (Fig. 4).

### Ground surface temperatures

**Fig. 5:**
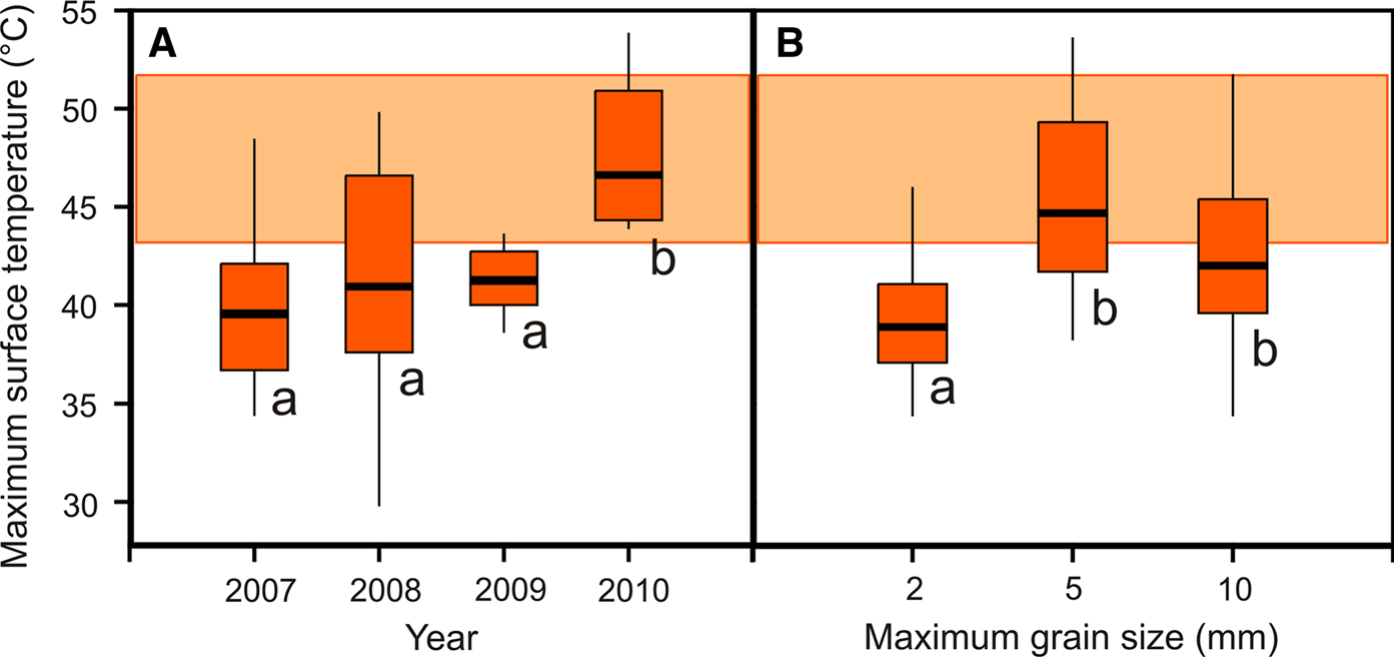
Maximum temperature (half an hour means) recorded at the ground surface (0–0.5 cm) during summer (June–July–August) at the pioneer stage of the Rotmoos glacier foreland (2,400 m asl). **a** Maximum temperatures differed between the years (2007–2010). **b** Maximum temperatures were affected by grain size: on sand (<2 mm), maximum temperatures were lower than on microsites with gravel diameters <5 and <10 mm. *Horizontal light orange bar* gives the range of heat tolerances (LT_50_) of the investigated field-grown seedlings. *Different letters* indicate significant differences

As ground surface temperatures appear most critical for heat survival, these temperature records were further analysed. Interannual differences in maximum ground surface temperatures were highly significant (Supplement 2). 2010 was the hottest year within the investigation period. In 2010, 100 % of temperature sensors recorded temperature maxima that were in the range or even exceeded the heat tolerance range of field-grown seedlings (Fig. 5a). A minor heat load was observed in 2007, 2008 and 2009: in these years, on 23.1, 38.5 and 9.1 % of measurement spots, respectively, temperature maxima would have been high enough to cause heat damage (>43.2 °C). Throughout 2007–2009 at mean on 70.7 % of measured microsites, maximum temperatures were not harmful.

Grain size was also found to be a significant factor for temperature maxima (Fig. 5b). On sandy ground surfaces (<2 mm), ground surface temperature maxima hardly (16.7 %) extended into the heat tolerance range of field-grown seedlings. At microsites with gravel (<5 or <10 mm), most temperature maxima (63.2 %) measured in air between gravel were within the range where heat injury to seedlings occurs. Temperature maxima recorded by sensors tips positioned below gravel extended to a lesser extent (14.3 %) into the heat tolerance range.

**Fig. 6:**
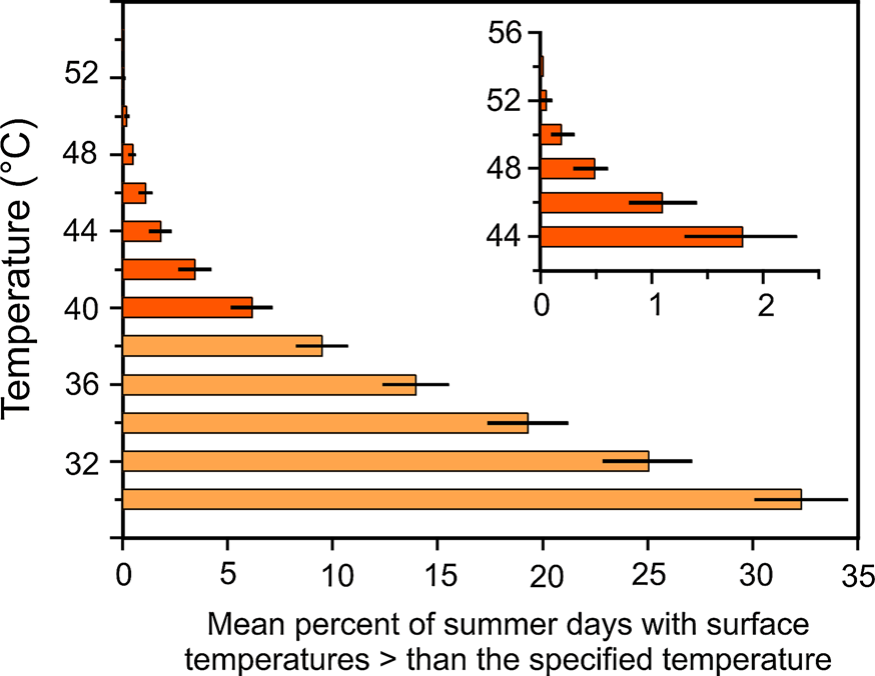
Mean per cent of summer days (% ±SE; June–July–August; 2007–2010) with ground surface (0–0.5 cm) temperature maxima (half an hour means) greater than the specified temperature. Ground surface temperatures were recorded at the pioneer stage of the Rotmoos glacier foreland (2,400 m asl). Temperatures potentially inducing heat damage in seedlings are coloured in a *darker orange color*

Overall warm (>30 °C) ground surface temperatures at the pioneer stage of the Rotmoos glacier foreland occurred at high frequency (>32.3 %) during summers 2007–2010 (Fig. 6). Critically high temperature maxima (30 min > 40 °C) occurred at a frequency of 6.2 %, in 2010 at 11.0 % of investigated days from June–August.

## Discussion

To our knowledge, these are the first results dealing with heat tolerance of early developmental stages of alpine plants. Imbibed seeds were found to be the most heat tolerant developmental stage investigated, followed by germinated seeds and seedlings. Still, dry seeds can survive higher temperatures (59–74 °C; Wildner-Eccher and Larcher, unpublished; cited in Neuner and Buchner 2012). With a heat tolerance of 55 °C (median), imbibed seeds must be considered to be hardly endangered by maximum temperatures recorded during the investigated years in the glacier foreland (highest half an hour mean value: 54.1 °C). Even seeds with lowest heat tolerance such as those of *Saxifraga aizoides* (range 47.3–53.0 °C) may be able to survive. The seeds of this species are the smallest ones in the glacier foreland (seed mass 0.04 mg, Schwienbacher and Erschbamer 2002; Schwienbacher et al. 2010). They can easily get buried and thus are able to avoid high temperature events at the ground surface. Together with most of the other glacier foreland species *Saxifraga aizoides* forms a persistent seed bank (Marcante et al. 2009b; Schwienbacher et al. 2010; 2011; 2012). Already at −0.5 cm soil depth, the severity of heat is considerably decreased. Heat stress might be a problem for seeds not forming a persistent seed bank, such as those of *Oxyria digyna, Geum reptans* and *Persicaria vivipara* (Marcante et al. 2009b; Schwienbacher et al. 2010). However, heat tolerance of seeds of these species was found to be higher 53.2 °C (*Oxyria*), 51.7 °C (*Geum*) and 56.5 °C (*Persicaria*). All in all, in the glacier foreland although not completely excludable, imbibed seeds appear to have a low risk of being lost by heat stress.

With the onset of germination, the vulnerability to heat increases. In laboratory grown seedlings, median heat tolerance was decreased by 4 K in germinated seeds and by 9 K in seedlings. Summer frosts were already outlined as major constraint in alpine environments (Körner 2003; Marcante et al. 2012). Our results add heat as a possible impeding environmental factor. Intraspecifically seedlings with a higher and lower heat tolerance can be differentiated, *Poa alpina* (52–55 °C) and *Trifolium pallescens* (50–51 °C) being the most heat tolerant species. *Poa alpina* was identified as ubiquitous species in the glacier foreland (Marcante et al. 2009a; Winkler et al. 2010) with seedling establishment along the whole glacier foreland, but with highest dominance at the early successional stage (Raffl et al. 2006; Schwienbacher et al. 2012). Grasses are generally predicted to be favoured by climate change (Walker et al. 2006; Shevtsova et al. 2009), probably also because of their generally increased heat tolerance (Buchner and Neuner 2001; Neuner and Buchner 2012) that is here corroborated for their seedlings. The comparatively high heat tolerance contributes to the explanation of species’ success along the successional sequences. The same holds for *Trifolium pallescens* that approaches quite fast recently deglaciated areas. Among more heat susceptible seedlings, the pioneer species *Saxifraga aizoides* showed tolerance minima of 34 °C, but also maxima up to 56 °C. In the arctic tundra, *Saxifraga cernua* exhibited a negative response to an artificially created heat pulse (temperatures above 44 °C, Graae et al. 2009). Even stronger negative effects of a heat pulse were shown for *Persicaria vivipara* (Graae et al. 2009). In our experiment, this species showed a mean tolerance of 43 °C.

Mean heat tolerance of field-grown seedlings (LT_50_; 46.1–50.7 °C) was found to be in the range of heat tolerance reported for adult alpine plants (vegetative parts: LT_50_ 42.0–64.0 °C compiled in Neuner and Buchner 2012; reproductive parts: LT_50_ 43.6–51.0 °C Ladinig, Neuner and Wagner unpubl.). For *Oxyria digyna*, *Poa alpina*, *Geum reptans* and *Silene acaulis*, heat tolerance data from adult individuals are available (Neuner and Buchner 2012). While *Oxyria digyna* and *Geum reptans* seedlings appear similarly heat tolerant as adults, *Poa alpina* and *Silene acaulis* seedlings showed a lower heat tolerance than reported for adults: 50.7 versus 56.2 °C and 47.2 versus 50.7 °C, respectively. It comes clear that developmental changes in heat tolerance need not be neglected when the establishment of plants is investigated.

It is also necessary to consider all organs as survival often depends on the most susceptible plant organ. Interestingly for the seedling, the cotyledons were detected to be the most heat susceptible ones, although they expand in the ground surface stratum where they get exposed to the most extreme temperature maxima. Still, cooling of cotyledons by transpiration may suffice to allow survival of heat. However, transpiration relies on a sufficient water supply that is successively reduced during sunny and hot periods. In alpine zones, the topsoil is known to desiccate even in the wettest mountains (Körner 2003). The topsoil is in turn the stratum were seedling roots initially may take up their water and in the pioneer stage top layers of undeveloped soils may additionally have only a poor amount of capillary water. Hence, water shortage and consequent reduced transpiration during hot days must be considered to be highly probable at the pioneer stage. Roots and hypocotyl were more heat tolerant and can be expected to better survive the moderated temperatures in the upper ground strata. Still, after heat damage to cotyledons, the recuperation capacity from the remainder parts, although not completely impossible, must be considered rather low.

Heat tolerance of seedlings cultivated in the growth chamber was significantly lower in six out of eleven species (at mean lower by 2.0 K). In seedlings, cultivated in the growth chamber minimum heat tolerance could be as low as 33.5 °C (*Saxifraga* *aizoides*). Minimum heat tolerance was in 6 out of 11 species lower than the lowest heat tolerance ever reported for vegetative parts of adult alpine plants (42 °C; Neuner and Buchner 2012). This demonstrates that for ecological interpretations, tests on field-grown plants are inevitably necessary. This intraspecific variability of heat tolerance between seedlings cultivated in the growth chamber and that grown at the field site is a consequence of heat hardening. In nature on alpine sites, heat hardening can occur at high rates 4.7 K/d within the species-specific heat tolerance range (6–11.7 K, Buchner and Neuner 2003). Heat hardening is observed when critically high temperature thresholds are surpassed (Alexandrov 1977). Threshold temperatures for the onset of heat hardening in higher plants are temperatures exceeding 30–32 °C (e.g.: *Silene acaulis*, Neuner et al. 2000). In the pioneer stage of the Rotmoos glacier foreland such temperatures (>30 °C) frequently (32.3 %) occur on the ground surface were seedlings establish. This may explain our result of a mostly increased heat tolerance of field-grown seedlings and emphasizes the necessity of field studies.

For survival and persistence of seedlings, microsites (Titus and Tsuyuzaki 2003; Chad and del Moral 2005; Akhalkatsi et al. 2006; Mayer and Erschbamer 2011) or safe site regeneration niches (Bell and Bliss 1980; Körner 2003; Shevtsova et al. 2009) are essentially necessary and offer in fine-scale environments so-called microtopographic shelters (Batllori et al. 2009). Our ground surface temperature records highly corroborate these fine-scale environmental differences in temperatures on the ground surface. Shading by a small stone can mitigate the temperature maximum significantly. Depending on the microstructure of the ground surface (sand or gravel) and the positioning of temperature sensors (in air or below small stones: 0–0.5 cm), maximum temperatures in small distance could vary between 31.9 and 49.3 °C (mean of 2007–2010). By this roughness of the soil, surface does not only govern deposition of seeds (Chambers et al. 1991; Chambers 1995) but also offers safe sites for germination (Chambers et al. 1990). Our results nicely demonstrate that microsites protected from heat can potentially promote survival. Similarly, facilitation effects by established plants were highlighted (Callaway et al. 2002; Elmarsdottir et al. 2003; Cavieres et al. 2005; 2006; 2007), creating “thermally stable sites” (Cavieres et al. 2007). Also in the glacier foreland, colonization depends on facilitation by established plants or stones (Niederfriniger Schlag and Erschbamer 2000; Erschbamer et al. 2008). Our results confirm that small stones are able to reduce maximum temperatures below them which may in certain situations suffice to survive heat. In contrast, the air between larger particles (<5 and <10 mm) is wind sheltered which reduces convection and causes considerable overheating to potentially critically high lethal temperatures. Chambers (1995) clearly showed that alpine seedlings were able to emerge at particle sizes of <0.5 to 4–8 mm. However, none of these seedlings survived in particle sizes larger than 1–2 mm, i.e. establishment seems to be possible only in fine-grained substrate. According to the author, larger particle sizes failed to provide ‘biological requirements’. We suggest that this could be due to overheating of air between larger particles.

Overheating of the ground surface was particularly pronounced in 2010, indicating significant differences between observation years. The heat wave of 2010 has already been recognized (Barriopedro et al. 2011). Ground surface temperature maxima (half an hour means) exceeded the lowest and highest heat tolerance of field-grown seedlings (43.2–51.7 °C) and reached a maximum of 54.1 °C. In 2010, temperature maxima were higher than 43.2 °C at all measurement spots at the ground surface 0–0.5 cm in air, but additionally on 80 % of microsites 0–0.5 cm where temperatures were recorded below a small stone. Until present, negative effects of heat waves on plants have scarcely been reported for alpine ecosystems (De Boeck et al. 2010, 2011, Abeli et al. 2012). Nevertheless, for vegetative above-ground plant parts of alpine species, heat damage has been observed to occur naturally at alpine field sites when after several sunny days water shortage reduces transpirational cooling (Larcher and Wagner 1976; Neuner et al. 1999; Buchner and Neuner 2003; Gauslaa 1984; Körner 2003). Water supply to seedlings on the undeveloped soils in the glacier foreland is poor (Schwienbacher et al., unpubl.). Generally, moist microenvironments could counteract overheating of the soil surface. However, the sandy ground material of the study site dries out quickly. Drought and resultant consequences appear to be the most important inhibitors of seedling recruitment and survival (Schwienbacher et al., unpubl. data). During sunny periods, lacking water availability in the upper soil strata may reduce transpirational cooling bringing seedlings even into higher risk of heat damage. Without transpiration, leaves tend to overheat. Even without overheating, our ground surface temperature records per se indicate a considerable risk of heat damage to seedlings in alpine glacier forelands due to the dryness of the substrate.

## Conclusions

The results of our experiment suggest that heat likely affects the recruitment of seedlings under water shortage in pioneer stages of glacier forelands, whereas heat effects on imbibed seeds may have comparatively minor importance. The heat tolerance during and after germination does not suffice to survive summer heat waves (i.e. events predicted to be more and more frequent in the course of climate warming) at the bare ground surface when water availability is reduced. Heat may at least partly explain the high seedling mortality recognized in recently deglaciated terrain of the glacier foreland. Locally, seedlings may profit from microenvironmental mitigation of ground surface heat caused by small stones or other safe sites that mitigate heat by shading, but additionally may provide enough water allowing transpirational cooling.

## Electronic supplementary material

Below is the link to the electronic supplementary material.

Supplementary material 1 (DOC 97 kb)
