# Supplementary material for: Heat tolerance of early developmental stages of glacier foreland species in the growth chamber and in the field
Source: Plant Ecol. 2014 Jul 2;215(7):747–58. doi: 10.1007/s11258-014-0361-8 (PMC4457355; doi:10.1007/s11258-014-0361-8)
Supplement: Supplementary file 1 — Supplementary material 1 (DOC 97 kb) [file 11258_2014_361_MOESM1_ESM.doc]

**Supplement 1** Heat tolerance (LT_50_) of the investigated glacier foreland species of different successional and developmental stages. Seedlings cultivated in growth chambers are compared to field grown individuals. In the seedling stage heat tolerance of cotyledons, the hypocotyl and the roots are additionally compared.

|  |  | Growth chamber grown |  |  |  |  |  | Field grown | | | | | | | | | |
| --- | --- | --- | --- | --- | --- | --- | --- | --- | --- | --- | --- | --- | --- | --- | --- | --- | --- |
| Species | Developmental stage | Individual  Mean ± SE | min | max | Cotyledones  Mean ± SE | min | max | Hypocotyl  Mean ± SE | min | max | Roots  Mean ± SE | min | max | Individual  Mean ± SE | min | max | ΔT |
| *SAXAIZ* | G1  G2  G3 | 49.3±0.7  49.6±0.9  44.6±2.4 | 47.3  46.4  33.5 | 53.0  56.7  55.7 | 45.3±0.06^A^ | 45.3 | 45.3 | 48.8±0.5^B^ | 47.3 | 49.3 | 47.3±0.06^C^ | 47.3 | 47.3 | 46.6±0.2 | 46.1 | 47 | 2.0^ns^ |
| *ARTGEN* | G1  G2  G3 | 55.9±0.3  44.8±0.7  45.3±0.8 | 54.9  41.0  41.0 | 56.9  47.0  46.9 | 48.6±0.6 | 46.7 | 49.6 | 51.1±0.5 | 49 | 51.5 | 51.5±0.01 | 51.5 | 51.5 | 50.4±0.02 | 50.3 | 50.5 | 3.8^**^ |
| *OXYDIG* | G1  G2  G3 | 53.2±1.2  50.2±0.7  46.9±0.5 | 47.3  46.9  44.7 | 56.4  53.0  49.0 | 47.4±0.5 | 44.7 | 48.5 | 45.9±0.7 | 44.7 | 51.5 | 47.7±0.5 | 44.7 | 49 | 47.9±0.5 | 44.7 | 48.9 | 1.0^*^ |
| *ARACOE* | G1  G2  G3 | 55.1±0.6  -  40.6±0.6 | 52.6  -  35.1 | 56.8  -  41.1 | 48.5±0.11 | 48.5 | 48.5 | 51.5±0.01 | 51.5 | 51.5 | 48.5±0.02 | 48.4 | 48.5 | 49.2±0.01^***^ | 48.9 | 49.6 | 8.7^***^ |
| ***Pioneer successional stage*** | **G1**  **G2**  **G3** | **53.4±0.5**  **48.2±0.6**  **44.3±0.7** | **47.3**  **41.0**  **33.5** | **56.9**  **56.7**  **55.7** | **47.4±1.6** | **44.7** | **49.6** | **48.3±2.9** | **44.7** | **51.5** | **48.6±1.9** | **44.7** | **51.5** | **48.5.±0.3** | **44.7** | **50.5** | **4.2** |
| *TRIPAL* | G1  G2  G3 | 52.8±1.1  49.5±0.8  50.2±0.1 | 47.3  47.0  49.8 | 56.4  53.6  50.5 | 48.0±0.05 | 48.0 | 48.0 | 50.8±0.05 | 50.8 | 50.8 | 50.5±0.05 | 50.5 | 50.5 | 50.2±0.1 | 49.8 | 50.5 | 0.0^ns^ |
| *PERVIV* | G1  G2  G3 | 56.5±0.2  51.3±0.0  43.4±0.2 | 55.1  51.2  43.2 | 56.7  51.4  43.7 | 46.8±0.7 | 45 | 49.2 | 47.8±0.4 | 46.7 | 49.2 | 53.4±0.2 | 52.9 | 53.8 | 49.1±0.9 | 44.6 | 51.7 | 5.7^*^ |
| *POAALP* | G1  G2  G3 | 50.9±0.0  52.5±0.3 | 50.9  51.7 | 50.9  55.0 | 49.6±0.05 | 49.6 | 49.6 | 53.8±0.05 | 53.8 | 53.9 | 51.5±0.05 | 51.5 | 51.5 | 50.7±0.08 | 50.3 | 50.9 | -1.8^***^ |
| *GEUREP* | G1  G2  G3 | 51.7±1.0  51.5±0.1  46.6±1.3 | 46.8  50.8  41.9 | 53.5  51.8  51.4 | 49.7±0.5 | 48.5 | 51 | 49.9±0.6 | 48.5 | 51.5 | 47.5±0.4 | 46.7 | 49.2 | 48.3±0.2 | 47.7 | 50.4 | 1.7^ns^ |
| ***Early successional stage*** | **G1**  **G2**  **G3** | **53.7±0.6**  **50.8±0.2**  **49.2±0.7** | **46.7**  **47.1**  **42.0** | **56.7**  **53.6**  **54.9** | **48.5±1.7** | **45** | **51** | **50.1±2.3** | **46.7** | **53.9** | **50.7±2.4** | **46.7** | **53.8** | **49.4±0.3** | **44.6** | **51.7** | **0.2** |
| *LEOHIS* | G1  G2  G3 | 54.4±0.4  50.2±0.2  44.6±0.9 | 53.0  49.5  38.5 | 56.9  51.0  49.5 | 44.6±1.4 | 42.1 | 47.3 | 49.1±0.7 | 48.5 | 51.1 | 47.3±0.8 | 45.9 | 49.9 | 46.1±0.9 | 43.2 | 48.3 | 1.5^ns^ |
| *ACHMOS* | G1  G2  G3 | 54.0±0.6  48.3±0.3  43.1±1.0 | 51.8  47.6  34.9 | 55.7  49.0  47.0 | - | - | - | - | - | - | - | - | - | 46.3±0.6 | 45 | 47 | 3.2^*^ |
| *SILACA* | G1  G2  G3 | 54.4±0.4  50.6±0.9  45.2±0.7 | 52.8  47.5  41.1 | 55.5  53.5  48.0 | 46.5±0.7 | 45 | 47.2 | 50±0.05 | 50 | 50.1 | 51.5±0.05 | 51.5 | 51.5 | 47.2±0.3 | 46.3 | 47.7 | 2.0^*^ |
| ***Late successional stage*** | **G1**  **G2**  **G3** | **54.2±0.2**  **49.9±0.4**  **44.3±0.5** | **51.8**  **47.5**  **34.9** | **55.7**  **53.5**  **49.5** | **45.3±1.8** | **42.1** | **47.3** | **49.2±1** | **48.5** | **51.1** | **48.9±2.1** | **45.9** | **51.5** | **46.6±0.4** | **43.2** | **48.3** | **2.3** |

Significant differences between vegetative and reproductive shoots are indicated by * (*P ≤ 0,05*), ** (*P ≤ 0,01*), *** (*P ≤ 0,001*), *ns* – not significant.

**Supplement 2** Determinants of maximum ground surface temperature (0-0.5 cm; half an hour means) at the pioneer stage of the glacier foreland as determined by GLM.

|  | | | | | |
| --- | --- | --- | --- | --- | --- |
|  | | | | | |
| Origin | Square sum type III | df | Mean of squares | F | Sig. |
| corrected modell | 1587,303^a^ | 26 | 61,050 | 3,940 | ,000 |
| constant term | 45074,827 | 1 | 45074,827 | 2908,969 | ,000 |
| year | 437,893 | 3 | 145,964 | 9,420 | ,000 |
| distance from ground surface (cm) | 331,836 | 3 | 110,612 | 7,139 | ,000 |
| Grain size (gs) | 210,436 | 2 | 105,218 | 6,790 | ,002 |
| year * cm | 87,077 | 7 | 12,440 | ,803 | ,589 |
| year * gs | 101,419 | 6 | 16,903 | 1,091 | ,381 |
| cm * gs | ,032 | 1 | ,032 | ,002 | ,964 |
| year * cm * gs | 22,148 | 3 | 7,383 | ,476 | ,700 |
| error | 759,261 | 49 | 15,495 |  |  |
| total | 129905,840 | 76 |  |  |  |
| Corrected total variation | 2346,564 | 75 |  |  |  |
| a. R-square = .676 (corrected R-square = .505) | | | | | |
